# Radiation therapy planning with photons and protons for early and advanced breast cancer: an overview

**DOI:** 10.1186/1748-717X-1-22

**Published:** 2006-07-20

**Authors:** Damien C Weber, Carmen Ares, Antony J Lomax, John M Kurtz

**Affiliations:** 1 Department of Radiation Medicine, Paul Scherrer Institute, Villigen-PSI, Switzerland; 2 Department of Radiation Oncology, Geneva University Hospital, Switzerland

## Abstract

Postoperative radiation therapy substantially decreases local relapse and moderately reduces breast cancer mortality, but can be associated with increased late mortality due to cardiovascular morbidity and secondary malignancies. Sophistication of breast irradiation techniques, including conformal radiotherapy and intensity modulated radiation therapy, has been shown to markedly reduce cardiac and lung irradiation. The delivery of more conformal treatment can also be achieved with particle beam therapy using protons. Protons have superior dose distributional qualities compared to photons, as dose deposition occurs in a modulated narrow zone, called the Bragg peak. As a result, further dose optimization in breast cancer treatment can be reasonably expected with protons. In this review, we outline the potential indications and benefits of breast cancer radiotherapy with protons. Comparative planning studies and preliminary clinical data are detailed and future developments are considered.

## Background

Postoperative radiation therapy very substantially improves local control in the treatment of both early and locally-advanced breast cancer. Trial overviews indicate that for every four local failures prevented, one fewer death from breast cancer can be expected. However, this long-term benefit can be mitigated somewhat by excess mortality due to cardiovascular disease and secondary malignancies [1]. Although local radiotherapy limited to the breast or chest wall can usually be administered using simple planning techniques with minimal late toxicity, regional treatment including lymph nodal areas can expose non-target organs to substantial radiation doses. One of the principal goals of treatment planning is thus to reduce any potential negative consequences of radiotherapy on long-term morbidity and mortality. This represents a particularly difficult challenge in the setting of loco-regional radiotherapy.

In recent years, great advances have been made in the planning and delivery of radiotherapy, as well as the development of existing imaging modalities. Computerized planning systems in conjunction with modern imaging studies are routinely used in breast cancer treatments. Three-dimensional conformal radiotherapy and, more recently, intensity modulated radiation therapy (IMRT) are being implemented increasingly in clinical use [2-6]. The delivery of optimal dose conformation can also be achieved with protons. Proton beam therapy is characterized by remarkable depth-dose distributions that have a low to median entrance dose, followed by a unified high-dose region (Bragg peak region) in the tumor area, followed by a steep fall-off to zero-dose distal to the target. As a result, physical dose distributions with protons are both highly conformal and homogeneous. Several proton facilities are currently operating worldwide and many more are scheduled to open in coming years. Proton beam therapy, however, is more costly than conventional treatment, and any potential benefits must be assessed in the light of the associated costs to the health-care system. Although comparative treatment-planning studies have demonstrated the superior dose conformation achievable with proton beams, it remains unclear whether protons can achieve substantial clinical gains in cancer types other than ocular melanoma or skull-base tumors. The industry-driven enthusiasm generated by proton dose distributions should not be allowed to outpace the clinical data investigating efficacy and safety in specific tumor sites. This review details the different proton beam delivery systems, with special emphasis upon the technical challenges of producing and delivering proton treatment beams for breast tumors. Dose-comparison studies of proton and photon beam therapy for breast cancers are reviewed, preliminary clinical data are detailed and future development considered.

### Proton beam therapy: delivery systems and biologic effects

The beam delivery system is the technical component that lies between the cyclotron and the patient. This system monitors patient dose, generates the desired 3D dose distribution within the patient and may also provide dynamic monitoring of its beam spreading and range control functions (see *dynamic scanning *technique). Two beam line designs are commonly used for proton therapy [7]. The *scatter foil technique *utilizes beam-flattening devices, collimators, scatterers, and energy modulation devices in the beam line to obtain a homogeneous dose in the target and sharp lateral penumbra [8]. Additionally, for each proton field, an individual aperture and compensator is manufactured and positioned in the proton beam [9]. Compensators will conform the distal dose fall-off to the target volume. In essence, it is a passive delivery system that relies on multiple coulombic scattering within the scattering foil devices for lateral beam spreading. A disadvantage of passive spreading is the interdependence of beam range and field size [8]. As field size increases, the scattering foil thickness must increase accordingly, resulting in loss of maximum treatment range. Most of the proton treatment facilities employ this simple and reliable delivery system.

As opposed to photons, protons are charged particles and can be easily deflected by the action of magnetic fields under computer control [7]. This opens the possibility for *dynamic scanning*, which can provide beam spread-out modulation by magnetically scanning the protons, with external apertures and compensators to conform the dose distribution. In *dynamic scanning*, no inherent interdependence of beam range and field size is observed. The ultimate *dynamic scanning *system is *voxel scanning *('spot' or 'raster' scanning) [10-12], in which the beam is decomposed into multiple, three dimensionally distributed Bragg peaks, which completely cover the target volume. Each voxel is irradiated to the planned dose, and the beam is switched off while moving to the adjacent voxel (spot scanning) [13]. This system is currently used at the Paul Scherrer Institut (PSI). Another active delivery system is the *raster scan system *that is used for carbon-ion radiotherapy at the Gesellschaft für Schwerionenforschung mbH, Darmstadt, Germany [14]. This active scanning system is based on the continuous irradiation with a radiation pencil beam through the target volume. A Belgian manufacturer (Ion Beam Applications) is currently implementing this delivery system for clinical use in the Boston proton beam facility. At PSI 3D dose conformation is generated without the need of external devices. Potential disadvantages include loss of precise tissue inhomogeneity compensation and potential increase in the lateral dose fall-off for beams that are conformed without external apertures. Furthermore, quality assurance is a more complex process for dynamic systems. External apertures, compensators and modulator wheels can be readily coded and identified in passive systems, but higher technology is involved to monitor beam spot motion and field uniformity. Noteworthy, the secondary neutron dose given to the patient with this beam delivery method might be lower by a factor of 10, when compared to the scatter foil technique. Various dose comparative studies have shown undisputedly that protons, when compared to photons, administer a lower integral dose to the patient [15,16]. This integral dose may cause secondary cancers. The production of secondary neutrons by the proton beam could however increase this integral dose and thus abrogate substantially the advantage of proton beam therapy for breast cancer. As such, the neutron dose has to be kept as minimal as possible. With spot scanning, the neutron dose in the Bragg Peak region can reach 1% of the treatment dose, but in the non-target volume this dose is roughly 2 – 4 × 10^-3 ^equivalent-dose (sievert) per Gy with the spot scanning technique and can be considered negligible [17]. Secondary neutrons are produced as a result of patient and material located in the proton beam path interaction, respectively. Hence, the production of these particles is dependent on the design of the beam line. Improving it (particularly the design and geometry of the Gantry's nozzle) might however decrease substantially the neutron dose with the scatter foil technique (A Thornton, PTCOG 44, personal communication). The neutron issue has been recently assessed in a review on IMRT and proton beam therapy [18].

It must be emphasized that protons have biologic effects in tissue similar to those of the megavoltage photons used in conventional therapy. They are regarded as low linear energy transfer particles, unlike other non-conventional radiotherapy particles, such as neutrons or carbon ions. The Relative Biological effectiveness of protons is defined as the ratio of the dose of a reference beam (usually ^60^Co or 6 MV) required to produce a specific effect in a biological system to the physical dose of proton radiation required to produce the same effect [19]. Its value is not fixed, but for 70 – 250 MeV protons range typically form 0.9 to 1.9, with an accepted 'generic' value of 1.1 in clinical proton therapy [20]. Consequently, the equivalent ^60^Co photon dose is the proton dose multiplied by 1.1. This calculated dose is defined as the Cobalt Gray Equivalent (CGE) dose. On behalf of the International Commission on Radiation Units and Measurements and the International Atomic Energy Agency, a committee will submit a report on Prescribing, Recording and Reporting Proton beam therapy in early spring 2006. It is proposed that the unit of Gy-isoeffective will be designated Gy(I). The full report will be published early 2007 (Dan Jones, personal communication 2006).

### Rationale for proton beams for breast cancer therapy

Photon whole breast irradiation (WBI) with two tangential fields sometimes administers substantial dose to the lung and, for left-sided breast cancers, to portions of the heart. When regional irradiation is indicated, the dose administered to these and other organs-at-risk (OARs) can be substantially increased. For this reason, a mixture of photon and electron beams is often used to treat the internal mammary nodes. Because of the need to match the electron and photon fields, this technique is characterized by considerable target dose inhomogeneity. Moreover, photon-beam irradiation of axillary lymph nodes also produces substantial dose inhomogeneities regardless of the technique used [21]. Newer radiotherapy techniques have permitted dose delivery to be conformed more precisely to the target volume. Tangential IMRT improves the dose homogeneity of WBI and reduces the dose to the heart or lung [2,3]. Similarly, IMRT techniques can improve homogeneity of dose delivery to the chest wall and internal mammary nodes for post-mastectomy radiotherapy, albeit at a cost of an increased dose to portions of the contra-lateral lung and breast [4]. Additionally, IMRT may decrease the administered dose to the abdominal organs when compared with conventional radiotherapy using physical wedges [6]. Using automated beam orientation and modality selection (electrons *vs*. IMRT), modulated electron radiotherapy has also resulted in an increased dose sparing to OARs with a somewhat less homogeneous target-dose delivery when compared to photon beams only [22]. Proton planning can also result in unparalleled homogeneous dose distributions within complex target volumes, while simultaneously sparing neighboring OARs. Comparative treatment planning studies have shown consistently that proton beam therapy can substantially decrease dose to OARs for various tumors [23-29]. This radiation modality could thus be delivered for the treatment of early or locally-advanced breast cancers. This review discussed several potential indications for the use of proton beams in breast cancer therapy.

## Methods

This review is based on Medline and PubMed literature searches using the key words 'breast neoplasm', 'radiotherapy', 'proton beam therapy', and the authors' clinical experience.

### Whole breast and loco-regional irradiation with protons

Meta-analyses of available randomized data by the Early Breast Cancer Trialists Collaborative Group have shown that radiation therapy decreases local recurrence rates by about 70% compared with surgery alone [1]. Absolute reductions of around 5% in 15-year breast-cancer mortality have been demonstrated both for patients treated with breast irradiation following conservation surgery and for node-positive patients treated with loco-regional irradiation following mastectomy. Although irradiation limited to the breast has not been shown to be associated with excess intercurrent mortality, about 1% more deaths due to causes other than breast cancer were observed among patients having receiving loco-regional post-mastectomy radiotherapy. This excess mortality was principally due to cardiac and other vascular causes, and to a lesser extent to secondary malignancies, particularly pulmonary [1]. An increased incidence of contralateral breast cancers was also observed in irradiated patients. Photon radiotherapy has also been associated with a small but incremental increase of long-term risk of contralateral breast cancer in a large SEER series [30] and data stemmed from randomized trials (Early Breast Cancer Trialists' Collaborative Group overview) [1]. Interestingly, the use of techniques that minimize cardiac dose, such as the use of electron beams to treat the mammary nodes and the chest wall, have been specifically used in two more recent post-mastectomy trials [31,32]; these particular studies do not show any deleterious effect of radiotherapy on cardiovascular mortality. These considerations demonstrate that maximizing dose sparing to the heart, or other OARs, such as the lung and contralateral breast, is of paramount importance both in early and locally-advanced breast cancer.

**Figure 1:**
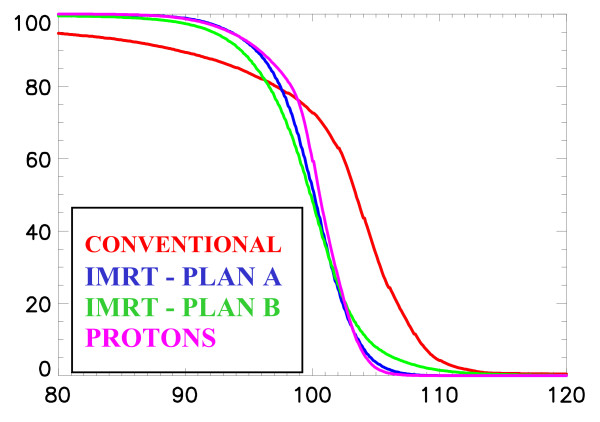
Cumulative dose-volume histograms for the conventional photon (Conventional), the intensity modulated treatment (IMRT 1–2) and the proton (Protons) plans for the breast and the breast and regional lymph nodes [33].

**Figure 2a:**
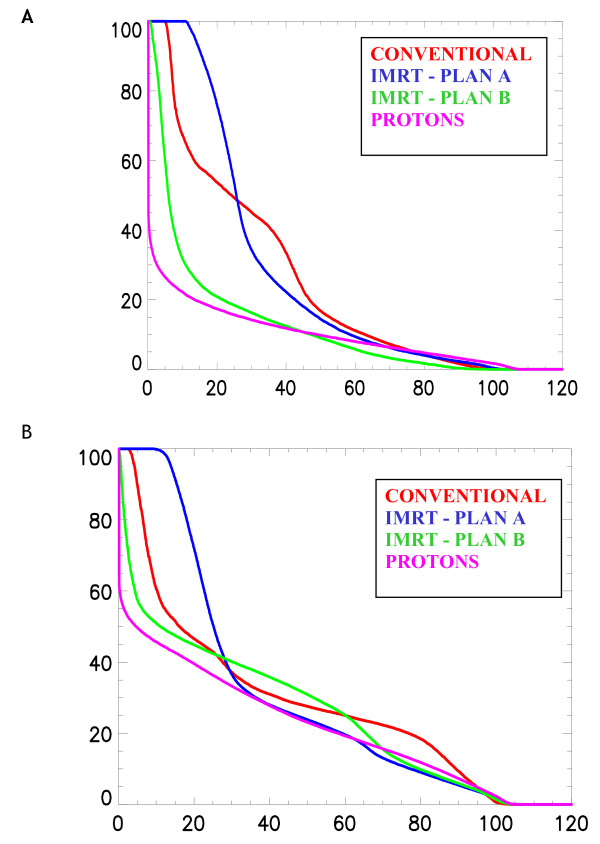
Cumulative dose-volume histograms for the conventional photon (Conventional), the intensity modulated treatment (IMRT 1–2) and theproton (Protons) plans for the heart [33]. **(B) **Cumulative dose-volume histograms for the conventional photon (Conventional), the intensity modulated treatment (IMRT 1–2) and the proton (Protons) plans for the ipsilateral lung [33].

In the irradiation of breast and regional lymph nodes, we have previously shown that protons, when compared to conventional or IMRT, deliver a highly homogeneous treatment with a substantial decrease of the mean dose delivered to the heart and contralateral lung alike [33]. In the PSI study, a two-field spot-scanned proton (left and anterior oblique fields), 9-fields (coplanar) IMRT (15 MV) and conventional plans (wedged 6 MV opposed tangential fields with anterior field to treat the internal mammary nodes using 26 Gy with 6 MV photons and 24 Gy with 12 MeV electrons) were computed and compared for a breast cancer patient. Mean doses delivered to the ipsilateral lung and heart were lower with protons. Moreover, the dose delivered to the contralateral breast was substantially reduced with protons, when compared to IMRT. For this OAR, the average values of the mean and maximum doses were 0.02 – 1.4 and 8.0 – 21.6 CGE-Gy for the proton and IMRT planning, respectively. This can be observed in the dose-volume histogram of the planned target volume (Fig. 1) and the OARs in the vicinity of the target volume (Fig. 2a, 2b). Likewise, Johansson *et al*. [34] reported on 11 node positive left-sided breast cancer patients for which one proton, one IMRT and two conventional plans were computed, respectively, for each patient. Irradiation techniques consisted on one single lateral oblique beam (30°), 6-fields (coplanar) 6 MV photon beams and tangential beams, with or without electron fields, for the proton (passive delivery technique), IMRT and conventional plans, respectively. The target volumes included the remaining breast parenchyma, the internal mammary nodes, and the supraclavicular-axillary lymph node regions. The prescribed dose was 50 CGE-Gy. According to a normal tissue complication probability (NTCP) model, protons reduced the NTCP for heart by a factor of 4 and for the lung by a factor of >20, when compared to the best photon plans. Although radiation pneumonitis generally represents a relatively minor clinical problem, potentially reducing the cardiac mortality from 6.7%, with the tangential technique, to only 0.5% with protons is likely to be clinically relevant, as a substantial number of patients, even those with positive nodes, will remain alive to be at risk for long-term morbidity [34]. Moreover, modern systemic adjuvant treatments, such as anthracycline-based chemotherapy, with or without taxanes, or trastuzumab [35], are associated with cardiotoxicity. High-dose delivery to the heart may further increase this risk in combination with these chemotherapy agents. Maximum heart distance and mean lung dose has been associated with cardiotoxicity in photon radiotherapy series [36]. IMRT significantly reduces the mean dose of the contralateral breast when compared to non-IMRT conventional tangential techniques [37], albeit at a cost of increased normal tissue radiation exposure [18]. Proton beam therapy further decreases the parasitic dose to the contralateral breast and nullifies the integral dose delivered to the patient [33]. Consequently, the implementation of radiation techniques that lower the integral dose of OARs in vicinity of the breast, such as protons, could be recommended for certain clinical situation (e.g., node positive left-sided tumors or inner tumor quadrant localization for young patients with large breasts).

Using biological parameters among other factors and a simple spot-scanned proton beam therapy technique (single-field), Fogliata *et al*. have demonstrated that protons reduce the lung equivalent uniform dose (EUD) significantly in both right- and left-sided tumors, when compared to other non-proton techniques (including IMRT) for postoperative whole breast radiotherapy [38]. Unlike the PSI [33] and Uppsala [34] study, the internal mammary chain, supraclavicular and axilla region was not part of the treatment volume for this planning-comparison exercise involving 5 patients with early breast cancer. Interestingly, the mean heart dose for the subset of patients with left-sided tumors was identical (mean, 2.6 CGE-Gy; range 2.2 CGE – 2.9 Gy). Maximum heart dose, however, was reduced with protons: a 40% absolute dose-decrease in hot spots was calculated with a single 100 MeV proton beam when compared to non-proton techniques. This derives from the heavily weighted heart-dose constraints applied to the optimization process of the IMRT planning with its consequential increased dose administered in the lung when compared to proton planning (lung volume receiving 20 CGE-Gy: 6% *vs *. 20% for protons and IMRT, respectively).

Table 1 details the planning target volume and doses administered to OARs for 17 breast cancer patients planned with protons and photons, with or without IMRT. On the average, 97% of the PTV receives 95% of the prescribed dose with protons compared to only 89% with conventional photon techniques. With protons, the mean dose to the heart is reduced by a factor of two to three when compared to photon planning, with or without IMRT. In these published studies proton plans have been calculated using only one [34,38] or two [33] fields. Such simple techniques could be easily used in a busy radiation oncology department. In contrast, for IMRT plans, sophisticated techniques were required in order to meet the planning goals and OAR's dose-constraints, resulting in an increased number (mean, 5) of beams. Overall, comparative planning studies have shown consistently that protons can reduce the administered dose to the heart, lung and contralateral breast in the treatment of breast with or without regional irradiation. It is possible that further proton dose optimization could be achieved by added proton field directions, resulting in an additional degree of dosimetric freedom.

**Table 1:** Overview of dose-volume histograms with proton, IMRT and photon conventional planning for the PTV and OARs in the proton-photon planning comparison literature

**PTV/OARs **Series (ref. no.)	V_95% _Protons (mean)	V_95% _IMRT (mean)	V_95% _Photons (mean)	Mean Dose (%) Protons	Mean Dose (%) IMRT	Mean Dose (%) Photons
PTV (breast only)						
Lomax *et al*. [33]	97.1	92.2	86.6			
Johansson *et al*. [34]	94.0	85.9	88.8			
Fogliata *et al*. [38]	99.8	95.5	92.2			
						
Heart						
Lomax *et al*. [33]				11.6	24.0	29.3
Johansson *et al*. [34]				21.0*	41.0*	61.0*
Fogliata *et al*. [38]				4.4	5.6	5.0
						
Lung (ipsilateral)						
Lomax *et al*. [33]				25.0	33.0	33.3
Johansson *et al*. [34]				1.0*	18.0*	29.0*
Fogliata *et al*. [38]				7.0	17.1	22.5

IMRT, intensity modulated radiotherapy; PTV, planning target volume; OAR, organ at risk; V_95%_, volume (in percentage) receiving 95% of the prescribed dose.

*estimated % of the prescribed dose from the dose-volume histograms administered to the heart and lung

### Partial breast irradiation with protons

**Figure 3:**
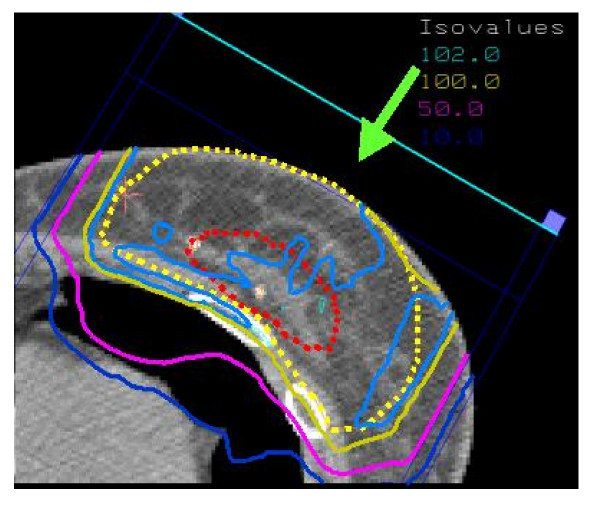
Dose distribution (protons) in an axial CT slice through the center of the breast for an early breast cancer patient treated with partial breast irradiation. The isodose contours are represented by different colors (corresponding values are displayed on the upper-right border of the figure).

Whole-breast irradiation with tangential photon beams is considered standard treatment following breast-conserving surgery. However, the inconvenience associated with conventional fractionation, and the substantial workload that breast cancer represents in busy radiation oncology departments, have led to increasing interest in other options for these patients. This subject has been reviewed elsewhere [39]. As most local failures after conservation surgery occur in the vicinity of the primary tumor bed, limiting the target volume to this area might achieve an acceptable degree of local control for selected patients whose tumors seem unlikely to be multifocal. The smaller irradiated volume may also more readily allow radiotherapy to be markedly accelerated, or even to be applied in a single fraction. This would substantially reduce the inconvenience associated with WBI, particularly for patients living far from treatment centers. Some of the acute and chronic toxicity of WBI might also be avoided, thereby improving patient satisfaction with treatment. Several retrospective accelerated partial breast irradiation (APBI) series [40-43] have appeared in the literature, and prospective randomized trials comparing WBI *vs*. APBI are ongoing (RTOG, GEC-ESTRO, Targit trial). APBI can be delivered using several techniques, namely low- and high-dose rate (HDR) brachytherapy using interstitial implantation [41,43-45] or a balloon catheter (MammoSite Radiation Therapy System; Cytyc Corp. Alpharetta, GA, USA) [46], 3D external beam conformal radiation therapy [47] or intraoperative radiotherapy (electrons or soft X-rays) [48,49]. Biologic comparison of APBI protocols has been recently reviewed [50]. Similarly with WBI, APBI could also be delivered using protons. Fig. 3 shows the dose distribution in an axial CT slice through the center of the breast using spot-scanning proton beam technology and a 1 field (direct) beam arrangement. This proton therapy planning was done on a patient treated at the Massachusetts General Hospital. The defined target volume consisted of the lumpectomy cavity plus a 20 mm margin. Taghian *et al*. have published the dosimetric comparison of APBI using protons with 3D conformal photon/electron based radiotherapy in 17 patients with early breast cancer [51]. PTV coverage for both modalities was equivalent. The maximum and median dose delivered to the heart, ipsilateral lung and non target breast tissue was however significantly decreased with protons for all patients. The Boston cohort has been recently updated and the initial clinical experience of 25 patients treated with APBI using proton beam therapy reported [52]. Using BID fractionation, 32 CGE was delivered to in 4 days, using 1 to 3 protons fields. To be enrolled in this phase I/II clinical trial, breast cancer patients had to have unifocal ≤2 cm tumors, negative margins (>2 mm) and pathologically negative axillary lymph nodes. The median volume of nontarget breast tissue receiving 50% of the prescribed dose was 23% and the median dose received by 5% of the ipsilateral lung was only 1.3 CGE. The controlateral lung and heart received essentially no irradiation. After observing acute moist desquamation at the treatment site in 3 patients treated with a single proton field, the treatment technique was refined and skin sparing was improved by the use of multiple (2–3) fields. These clinical data from Boston suggest that APBI using protons is technically feasible and provide optimal OAR sparing.

Similarly, proton beam therapy could be delivered for simultaneous integrated boost delivery (SIB) during WBI. Notwithstanding the importance of the boost delivery on local control [53,54], this additional radiation dose could be delivered not sequentially but concomitantly to the WBI. This would allow reduction of the overall treatment time by 1.5 – 2 weeks by delivering the boost to the tumor bed simultaneously with the whole breast schedule. Giving higher fractional boost doses (≈2.2 – 2.4 CGE-Gy/fraction) will administer higher biological equivalent dose (BED) to the target volume. As the dose distributions achieved with IMRT or protons are highly conformal, OARs (heart, lung) that are not directly surrounding the target regions will not receive a higher dose per fraction and are therefore not at greater risk for late toxicity. Furthermore, this type of concomitant boost schedule is a more efficient way of planning and radiation delivery as it involves the use of the same plan for the entire course of treatment. This SIB strategy is however a significant departure from conventional radiotherapy experience. Radiation therapy schedules are aimed at giving a high uniform dose to the target volume for every fraction and then reducing the volume to the boost portion. SIB has been mostly studied for head and neck and prostate cancers and occasionally for breast cancer in recent years [55]. A Stanford study, however, has demonstrated that a SIB-IMRT schedule for breast cancer increases the heart and lung volumes receiving low-dose irradiation, indicating that caution must be observed with regard to the OARs when attempting to escalate the target dose [56]. Such an increase in dose to the non-target breast tissue, heart and lung would not be observed with protons. It can be hypothesized that using a proton-SIB strategy, shorter biologically equivalent schedules could be calculated and possibly implemented in clinical use. If a planning target volume is defined by a 1-cm margin around the surgical cavity, the radiobiological aspects of such a strategy will be favorable, as only a limited volume of non-involved breast tissue (within the planning target volume) will be treated with a high fractional dose. Parenthetically, administering a higher fractional boost dose with protons can be achieved with or without intensity modulation. Using the spot scanning technology, which dynamically position Bragg peaks, differential weights could be individually defined within the target volume. This will allow using these dose spots (i.e. Bragg peaks) to 'paint' the dose as required with full flexibility. Theoretically, using intensity-modulated proton therapy (IMPT), with its ability to deliver fields of arbitrary complex fluence profiles, will probably result in more homogeneous dose deposition when compared to non-IMPT plans. This derives from the fact that the highly inhomogeneous individual IMPT fields, which when combined produces a homogeneous dose distribution, will compensate for the dose deposition of the other field's complex 3-D dose distributions in the optimization process. In other words, the IMPT plans will ultimately balance more evenly the high-dose regions around the target volume than could the non-intensity modulated protons. No proton-SIB data for breast cancer have been yet published. A radiobiological and treatment planning study for breast cancer is currently being carried out at PSI, comparing conventional schedules with IMRT- and proton-SIB treatments. These calculations could be useful as means of designing fractionation strategies for use in clinical protocols with SIB with or without protons.

Finally, protons could be used for sequential boost radiotherapy after WBI. Randomized trials have demonstrated that local control can be significantly improved by addition of a localized tumor-bed boost delivered following standard WBI [53,54]. In the large trial by the European Organization for Research and Treatment of Cancer the addition of a 16 Gy boost reduced the local failure rate by a factor of almost 2, compared with 50 Gy WBI alone, albeit at the cost of a greater number of fair-poor cosmetic results [57]. Although most patients received electron-beam boosts, results seemed similar using brachytherapy or external photon beams. It could be argued that the lateral dose fall-off may be an advantage with protons. As the mass of protons is larger, when compared to electrons, the angles of Coulomb interaction scattered particles are smaller. It could be counter-argued that a larger lateral dose fall-off could be however beneficial if the target volume is ill-defined, which is usually the case for the clinical planning of the boost. Additionally, the logistical problems associated with a proton-boost only delivery after breast radiotherapy with photons would be surely prohibitive. Protons will surely play a minor role, if any at all, in the development of sequential boost protocols.

All forms of partial breast treatment, namely, the APBI, sequential boost and SIB, using protons is surely a very effective means of limiting doses to normal structures, but this modality has a number of potential shortcomings that must be carefully considered. First, inter- and intra-fraction tumor motion may abrogate any ballistic advantage of protons and mitigate any potential clinical benefit. These motions during proton beam therapy can introduce substantial unplanned heterogeneities in the dose distribution throughout the target volume [58]. Specific methods of breast-dose delivery, similar to those implemented with photon radiotherapy [59], mitigating the effects of organ motion, should thus be actively pursued, such as breath hold and gating methods [60]. Second, the availability of proton beam therapy for this prevalent disease is questionable. Photons and electrons are available worldwide and have been used in this setting for many years unlike protons, which are restricted to a very few centers. Third, the excellent cosmetic results achieved with modern photon therapy will not be improved with protons, which do not deliver a lower skin dose when compared to electrons. As mentioned earlier, the initial superficial dose proximal to the target volume is generically 30 – 40% of the maximum prescribed dose. More specifically, for a superficial tumor, located 20 mm from the skin surface and a 20 mm diameter (pT1c), the percentage of the total dose delivered to this region would be 85 – 90%, using a 160 MeV direct proton beam. This compares identically with the electron dose deposition, where 95% of the total dose would be administered (applicator 10 × 10 cm) on the skin using a 6 MeV energy electron beam. In the phase I/II APBI clinical trial from Boston, the first 3 patients treated with one proton field experienced acute moist desquamation at the treatment site [52]. Subsequently, all patients were treated with a 2 – 3 fields treatment technique. As such, sophistication of the radiation technique using 1 proton field will not improve the cosmetic outcome unlike photon radiotherapy for which cosmesis was indeed favorably influenced by improved technical factors in radiation delivery in a recent series [61]. Finally, the production of secondary neutrons produced by nuclear interactions in the material in the beam line is a concern with proton beam therapy. The dose produced by these uncharged particles depends on the materials – geometry of the beam material delivery system and the energy of the primary proton beam [62]. Estimating the neutron dose by performing measurements and Monte Carlo simulations, Schneider *et al*. have demonstrated that the contribution to the integral dose from neutrons is very low (in the order of 2 × 10^-3 ^Sv per delivered Gy) using the spot scanning technique [17]. This neutron-integral dose contribution, however, could be much higher (by a factor of ten) using passive delivery systems, as a result of the various scatterers, beam-flattening devices, collimators and compensators that are hit by the primary proton beam. Thus, the proton's scatterer foil technique could substantially increase the high-LET neutron delivered integral dose, although this leakage neutron-radiation could be substantially decreased with improvement in the nozzle design. Such a nozzle-design modification has been undertaken at the Midwest Proton Therapy Center (Bloomington, IN, USA), with measured neutron doses substantially lower than those from other passive scattering delivering systems (Allan Thornton, personal communication, 2006). This additional dose with a large biological factor could however consequentially translate in an increase of radiation-induced cancers.

### Cost and availability of proton beam therapy

In the United States, the costs of breast conservative treatment are significantly higher than those generated by modified radical mastectomy, with or without breast reconstruction [63]. The addition of radiation therapy results in the higher costs of conservative surgery, representing roughly 70% of the total billing. Interestingly, Palit *et al*. reported that the physician's fee for radiotherapy were significantly higher than the surgeon's and amounted alone to roughly one-third of the total radiation therapy billing [63]. New technologies can contribute, at least theoretically, to reducing costs of breast cancer radiotherapy; for example, multileaf collimation virtually eliminates the need for beam blocking and reduces treatment time, and particle beam delivery systems reduce the number of treatment portals required [64]. In the majority of cases, however, emerging technologies will ultimately translate into increased total billing as a result of increased time dedicated to treatment planning and the obligate acquisition of new planning and delivery equipment, among other factors. In general, the additional cost factor for proton therapy over that for intensity-modulated photons is now 2.4 – 3.0 [65]. For most of the treatment planning and treatment, the costs for protons and photons are identical. The differential costs are accounted for by the proton accelerator and the engineering staff required for operating the facility. It is reasonable to assume that the expense of proton therapy per patient will decrease, as more facilities are built and greater numbers of patients treated. A substantial number of proton beam facilities are currently been planned and built worldwide [66]. In the US, these proton beam therapy facilities involve major cancer centers such as the M.D. Anderson Cancer Center, Houston TX, the Children's Hospital of Philadelphia, Philadelphia PA and the University of Florida College of Medicine, Gainesville FL, to name a few. Additionally, accelerated proton beam therapy schedules (e.g. APBI, SIB) may further decrease the treatment-related cost as shown recently in a clinical trial [52]. Cost analysis of the Boston cohort suggested that proton APBI was only modestly more expensive (25%) than traditional WBI with a sequential boost. It must be stressed that these direct costs do not account for other aspects of treatment, such as patient's satisfaction or quality of care. Interestingly, a cost-effectiveness analysis of proton radiation has been published by the Karolinska Institute group [67]. This group used a cohort-simulation mathematical model comparing two hypothetical cohorts of women with breast cancer receiving either proton beam therapy or conventional irradiation. The Markov-model simulated the course of events in individual patients from diagnosis until death or until age 100 years. Individuals were modelled in differential health states, each associated with a certain cost and utility. In this study, proton beam therapy provided an incremental benefit for an average breast cancer patient. The costs and quality adjusted-life years gained was estimated to €67,000 for proton beam therapy. Base-case simulation suggested that a 2.4% and 13% decrease of fatal cardiac disease and pneumonitis, respectively, should be observed with protons when compared to conventional irradiation. These data suggests that proton beam therapy can be cost-effective and cost saving for specific breast cancer indications, when compared to conventional radiotherapy. We now appear to be heading to a watershed where an increased therapeutic index and cost-effectiveness of protons come together. Although not formally studied in a clinical setting, it is reasonable to hypothesize that the use of proton beam therapy for high-risk breast cancer patients could translate into less late radiation-induced toxicity, thus improving the overall quality of care for these patients. Likewise, decreasing the acute side effects of radiotherapy will promote the physical well-being and early return to occupational/social activities after treatment. Similarly, the administration of APBI or SIB with protons could potentially decrease the overall-treatment time and thus improve the patient's burden associated with the long course of radiotherapy. The perception that proton radiation therapy is less cost-effective than non-proton radiotherapy in specific clinical situations may be challenged by the potential for improvements in clinical outcomes for advanced breast cancer patients with extensive nodal involvement requiring regional radiotherapy or shortened adjuvant radiation courses (e.g. APBI or SIB) for early breast cancers.

## Conclusion

Based on the analysis presented in this paper, we believe that proton irradiation may have some potential for improving the outcome for patients with early and high-risk patients alike. However, the increased cost factor and the questionable availability of protons for such a common disease could seriously hamper their routine use for breast cancer. Substantial additional research will be required before a role for proton therapy in this setting can be established. Using the methodology of dose-comparison analysis, the impact of protons on dose deposition for certain clinical situations should be more thoroughly assessed, and the functional effects of dose sparing to OAR's should be formally investigated.

## Abbreviations

**IMRT**, intensity modulated radiotherapy; **PSI**, Paul Scherrer Institut; RBE; **CGE**, Cobalt Gray Equivalent; **Gy(I)**, Gy-isoeffective; **WBI**, whole breast irradiation; **OAR**, organ at risk; **NTCP**, Normal Tissue Complication Probability; **EUD**, equivalent uniform dose; **APBI**, accelerated partial breast irradiation; **BED**, biologic equivalent dose; **SIB**, simultaneous integrated boost; **IMPT**, intensity-modulated proton radiation therapy; **Sv**, Sievert.

## Competing interests

The author(s) declare that they have no competing interests.

## Authors' contributions

DCW conceived and wrote the review, CA, AJL and JMK reviewed the manuscript.
